# New Insights into Anthelmintic Mechanisms of Action of a Synthetic Peptide: An Ultrastructural and Nanomechanical Approach

**DOI:** 10.3390/polym13142370

**Published:** 2021-07-20

**Authors:** Alexandra M. S. Soares, Luciana M. R. Alencar, Lucas P. Dias, Ruana C. Lima, Carolina Rocha Silva, Ralph Santos-Oliveira, Jose T. A. Oliveira, Livio M. C. Junior, Pedro F. N. Souza

**Affiliations:** 1 Laboratory of Plant Biochemistry, Federal University of Maranhão, São Luís 65080-805, MA, Brazil; alexandra_soares@ufma.br; 2 Biodiversity and Biotechnology Graduate Program-BIONORTE, São Luís 65080-805, MA, Brazil; carolinars@live.com; 3 Laboratory of Biophysics and Nanosystems, Federal University of Maranhão, São Luís 65080-805, MA, Brazil; luciana.alencar@ufma.br (L.M.R.A.); ruanalima1@gmail.com (R.C.L.); 4 Laboratory of Plant Defense Proteins, Biochemistry and Molecular Biology Department, Federal University of Ceará, Av. Mister Hull, P.O. Box 60451, Fortaleza 60020-181, CE, Brazil; lpinheirodias@gmail.com (L.P.D.); jtaolive@ufc.br (J.T.A.O.); 5 Laboratory of Parasite Control, Federal University of Maranhão, São Luís 65080-805, MA, Brazil; livio.martins@ufma.br; 6 Laboratory of Nanoradiopharmaceuticals and Radiopharmacy, Zona Oeste State University, Rio de Janeiro 23070-200, RJ, Brazil; presidenciaradiofarmacia@gmail.com; 7 Brazilian Nuclear Energy Commission, Nuclear Engineering Institute, Rio de Janeiro 21941-906, RJ, Brazil

**Keywords:** nematode, *Haemonchus contortus*, synthetic peptides

## Abstract

Resistant nematodes are not affected by the most common drugs commercially available. In the search for new anthelmintics, peptides have been investigated. Here, a linear synthetic peptide named *RcAlb*-PepIII bioinspired from the antimicrobial protein *Rc-2S-Alb* was designed, synthesized, and tested against barber pole worm *Haemonchus contortus*. The physicochemical properties of the peptide, the 3D structure model, the egg hatch inhibition, and larval development inhibition of *H. contortus* were carried out. Additionally, the ultrastructure of the nematode after treatment with the peptide was evaluated by atomic force microscopy. The *RcAlb*-PepIII inhibited the larval development of *H. contortus* with an EC_50_ of 90 µM and did not affect egg hatch. Atomic force microscopy reveals the high affinity of *RcAlb*-PepIII with the cuticle of *H. contortus* in the L2 stage. It also shows the deposition of *RcAlb*-PepIII onto the surface of the cuticle, forming a structure similar to a film that reduces the roughness and mean square roughness (Rq) of it. In conclusion, the bioinspired *RcAlb*-PepIII has the potential to be used as a new anthelmintic compound to control gastrointestinal nematode parasites.

## 1. Introduction

Infection caused by multi-drug resistant nematodes in ruminants led to a negative impact on livestock production and significant economic losses worldwide [1,2] (To face these parasitic infections, a diversity of anthelmintic drugs is available. However, parasite resistance has shown a rapid increase in the last years caused by the misuse or overuse of the conventional antiparasitic drugs, leading to ineffective treatments [3]. *H. contortus* is a hematophagous gastrointestinal nematode from a ruminant abomasum with the highest impact on livestock production. Several studies have reported multi-drug *H. contortus* resistance on strains of small ruminants’ farms from all continents worldwide [4,5,6]. Based on that, the development of new safe and efficient drugs to treat these infections is urgent. However, despite the urgency, the development of novel compounds for helminths has been slow [7].

Antimicrobial peptides are natural antibiotics produced by all living organisms, including plants, which are known to contain more than 273 compounds [8] (http://www.phytamp.hammamilab.org/main.php, accessed on 23 March 2021). They are part of the first line of defense toward microbes such as bacteria, fungi, and protozoa or viruses [9,10]. The anthelmintic effect of these compounds has been shown on free-live nematodes and human, canine, and ruminants gastrointestinal nematodes [11,12,13]. As a result of this bioactivity, the antimicrobial proteins and/or peptides from plants have gained attention as an alternative treatment for parasite infection. However, three problems arise: (1) high cost for the purification process; (2) low resistance to proteolysis; and (3) toxicity to host hidden practical applications [14]. As a solution for that, synthetic peptides have come out. Synthetic peptides are designed from antimicrobial protein sequences to improve the activity and resistance to proteolysis and reduce toxicity [14].

Our research group purified a potent antimicrobial protein from *R. communis* seed cake, *Rc-2S-Alb* [15], which was used to design synthetic bioinspired peptides named *RcAlb*-PepI, *RcAlb*-PepII, and *RcAlb*-PepIII. The synthetic peptides *RcAlb*-PepI and *RcAlb*-PepII showed antimicrobial activity against *Escherichia coli, Klebsiella pneumoniae, Staphylococcus aureus, Candida albicans, Candida parapsilosis,* and *Candida tropicalis* [16], but not against *H. contortus*. Here, *RcAlb*-PepIII has arisen as a potential anthelmintic peptide for having inhibitory effects on the larval development of *H. contortus*. Atomic force microscopy (AFM) revealed insights about the mechanism behind *RcAlb*-PepIII against the gastrointestinal nematode *H. contortus*, revealing the biotechnological potential of *RcAlb*-PepIII to develop peptide-based anthelmintic drugs. 

## 2. Materials and Methods

### 2.1. Peptide Design, Characterization by Bioinformatic Analysis, and Chemical Synthesis

The peptide *RcAlb*-pepIII was designed as previously described [16] based on the amino acid sequence of *Rc-2S-Alb* [15].

Predicting Antigenic Peptides (http://www.imed.med.ucm.es/Tools/antigenic.pl, accessed on 15 January 2021); Peptide Cutter tool (http://www.web.expasy.org/peptide_cutter/, accessed on 15 January 2021); and HLP (http://www.crdd.osdd.net/raghava/hlp/help.html, accessed on 15 January 2021) [17]. These tools are freely available on the Internet. The peptide *RcAlb*-PepIII was chemically synthesized by GenOne (São Paulo, Brazil), which analyzed their quality and purity (≥95%) by reverse-phase high-performance liquid chromatography (RP-HPLC) and mass spectrometry. The three-dimensional (3D) structures were produced using the PEPFold server [18].

### 2.2. Anthelmintic Assays

To this assay, the *RcAlb*-pepIII peptide was solubilized in the 50 mM PBS 7.4 pH buffer with a concentration ranging from 50 to 300 µM. Third-stage larvae (L3) of a monospecific strain of *H. contortus* (n = 2000 L3/animal) were used to experimentally infect two six-month-old lambs (Santa Inês breed), which were maintained in a metabolic cage. The infected lambs received hay and water ad libitum and 1% of its live weight of commercial feed with 20% crude protein. *H. contortus* eggs were obtained according to Coles et al. [19]. Experimental procedures were performed following the guidelines of the Animal Ethics Committee of Maranhão Federal University. This committee approved them under protocol number 23115018061/2011-11.2.3.2.

### 2.3. Egg Hatch Test (EHT)

Freshly collected feces were mixed in warm water (37 °C), and the eggs were recovered from the solution in 25-µm sieves. Afterwards, eggs were added to a saturated NaCl solution and centrifuged (3000× *g*) for 3 min; floating eggs were recovered using a 25 µm sieve. Eggs were washed three times to eliminate the remaining salt and re-suspended in distilled water. A serial dilution in PBS pH 7.4 was performed, with a final concentration of peptides ranging from 50 to 300 µM. In the egg hatch test (EHT), approximately 100 eggs per well were plated in 96-well flat-bottom plates, with four replicates, and the different dilutions were added. The plate was incubated at 27 °C and Relative Humidity (RH) >80%; after 48 h, the eggs and larvae were quantified under an inverted microscope [19].

### 2.4. Larval Development Test (LDT)

*H. contortus* eggs were obtained as previously described. To 96-well flat-bottom plates were added 100 eggs/well (100 µL), which were incubated at 27 °C for 24 h until the eggs reached the first-stage larvae (L1). Then, 40 µL of *Escherichia coli* medium (autoclaved *Escherichia coli*) at 0.15 mg mL^−1^, yeast extract at 8 mg mL^−1^ in saline 0.9%, Earle’s solution at 0.22 mg mL^−1^, and amphotericin B (Sigma A2942) at 0.018 mg mL^−1^ were added in each well. The peptides were diluted in PBS pH 7.4 (as discussed above), and 110 µL were added to reach six final concentrations among 50 to 300 µM. Four replicates of each concentration were performed. The 96-well plates were incubated at 27 °C for six days, and then, L1 and L3 were counted under an inverted microscope [20].

### 2.5. Atomic Force Microscopy (AFM) Analyses

These AFM measurements were performed for the characterization of *RcAlb*-PepIII and assessment of the topography and mechanical properties of larvae with and without treatment. The larval development assay was carried out as previously described using the peptide in the IC50 concentration. Phosphate buffer solution (pH 7.4) was used as control. Eight replicates of the experiment were carried out. After 48 h, the control was observed under an inverted microscope to confirm the second-stage larvae’s presence (L2). The content of four replicates and its control were collected and fixed in 10% paraformaldehyde (1:1). Then, 120 h after starting the test, the other wells containing L3 were fixed as described.

The samples of L2 and L3 *H. contortus* control and treated were deposited in 13 mm glass coverslips previously treated with poly-l-lysine 1% (Sigma, St. Louis, MO, USA), aiming to improve the substrate’s adhesion and avoid the samples being dragged during scanning. AFM measurements were performed using a Multimode 8 microscope (Bruker, Santa Barbara, CA, USA) in PeakForce Tapping Quantitative Nanomechanics mode, in air (23 °C temperature and 44% humidity), using silicon probes with a nominal spring constant of 0.4 N/m and nominal tip ratio of approximately 2 nm. However, the actual spring constant of each probe used in this work was measured by the thermal noise method. Images were taken at approximately the center region of the larvae, with a size of 1 µm × 1 µm for better visualization of peptides on larvae cuticles. The resolution of images was 256 × 256 force curves [21]. 

The roughness values were calculated from each pixel in the entire AFM height maps, using NanoScope Analysis Software 2.0 (Bruker) according to Equation (1):

(1) $$\sqrt{\frac{\sum z_{i}^{2}}{N}}.$$

That defines the root mean square average of height deviations taken from the mean image data plane, where *z* is each pixel height value and *N* is the total number of pixels (in our case 65,536). To avoid changes in the real values, no image pretreatment was performed on the maps before the calculation of this parameter.

The adhesion and stiffness data of the samples were calculated from each of the force curves obtained on all samples under study. For the calculation of the adhesion between the probe and the sample surface, the most negative value of force (cantilever deflection) obtained from the retraction curve was considered. These values represent the resistance of the AFM probe to leave the sample surface. For the calculation of the surface stiffness, the slope values in the contact portion of the approach curves were considered, in intervals of 30–70% of deflection after the contact point of the tip with the sample.

For the peptide characterization, 10 μL of *RcAlb*-PepIII (1 mg mL^−1^) was deposited in previously cleaved mica and analyzed in the AFM under the same conditions previously described. The solution was left in the mica until completely dry and then analyzed on the AFM. 

### 2.6. Hemolytic Potential and Toxicity to Vero Cell Lines of RcAlb-PepIII

To evaluate the hemolytic potential, the peptides (50, 100, 150, 300, and 600 μM) were incubated with rabbit red blood cells for 1 h at 37 °C [16]. Experimental procedures were performed following the guidelines of the Animal Ethics Committee of Maranhão Federal University and were approved by this committee under protocol number 23115018061/2011-11.2.3.2.

The toxicity of *RcAlb*-PepIII was assessed by cell viability assay using 3-(4,5-dimethylthiazol-2-yl)-2,5-diphenyltetrazolium bromide (5 mg mL^−1^, MTT) described by Souza et al. [14]. Cell viability (CV) was calculated as follows: CV (%) = Abs (treated)/Abs(control) × 100. The DMSO-NaCl solution was used as control.

## 3. Results

### 3.1. Design and Bioinformatics Data of RcAlb-PepIII

*RcAlb*-PepIII was designed from the primary structure of *Rc-2S-Alb* [15] using the C-PAmP tool [22] as an antimicrobial peptide. In this study, *RcAlb*-PepIII was described as an anthelmintic peptide. Based on that, some of the physicochemical and biological properties of *RcAlb*-PepIII were compared with Kalata b1, which is a well-known anthelmintic peptide [11]. Regarding physicochemical properties, both peptides are quite different. Starting at molecular weight, *RcAlb*-PepIII is smaller and basic (826.05 Da, pI 8.80) compared to Kalata b1 (2979.81, pI 4.7). *RcAlb*-PepIII has a higher hydrophobic potential and is positively charged, whereas Kalata b1 is negatively charged (Table 1).

**Figure 1:**
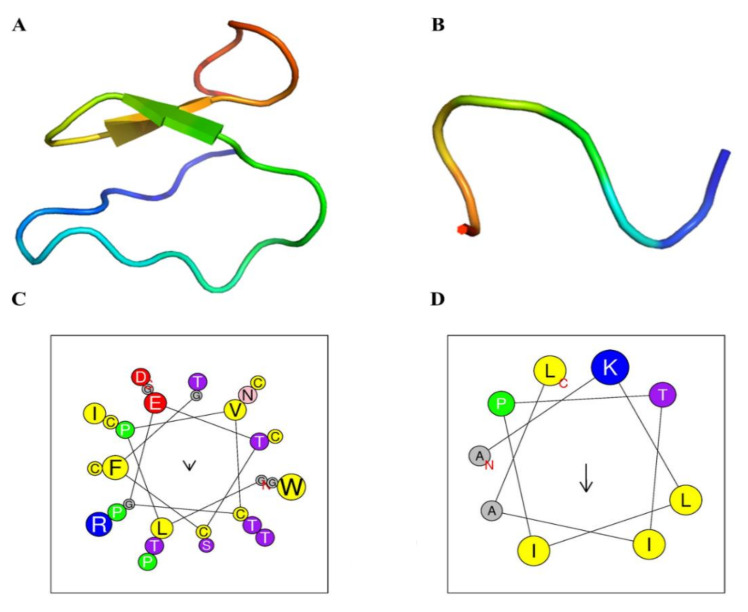
Three-dimensional (3D) structures and helical wheel projections of Kalata B1 and *RcAlb*-PepIII. Comparison between 3D structures of Kalata B1 (**A**) and *RcAlb*-PepIII (**B**) where both peptides presented unordered structures. The helical projection of Kalata B1 (**C**) and *RcAlb*-PepIII (**D**) revealed the higher complexity of the Kalata B1 structure compared to *RcAlb*-PepIII. The HeliQuest site was used for the construction of diagrams and projections and the PEPFold server to produce 3D structures. The hydrophobic residues are represented in yellow, apolar residues are in silver, while positively charged residues are in blue, and the polar residues are in violet and magenta. The arrows represent the helical hydrophobic moments.

Despite the differences in the physicochemical properties, both peptides have some structural similarities. *RcAlb*-PepIII, a three-dimensional model, was constructed using the PEPFold server, and a 3D model of Kalata b1 was downloaded from a protein data bank (PDB, 2KHB). It is clear and expected that Kalata b1 has a larger structure than *RcAlb*-PepIII (Figure 1). However, the 3D models are too similar. Both peptides have an unordered structure as predominant in the 3D model (Figure 1). Despite that, Kalata b1 possesses a minor part of the structure composed of beta sheets (Figure 1A). Wheel projection of both 3D sequences revealed that even though the hydrophobicity of Kalata b1 is lower than *RcAlb*-PepIII, it has a hydrophobic face composed of residues of Phe, Cys, Pro, and Cys (Figure 1C). The wheels project revealed a simple conformation of *RcAlb*-PepIII without a clear hydrophobic face (Figure 1D).

Biological properties analyses in silico brought to light new features that tell *RcAlb*-PepIII and Kalata b1 apart (Table 1). *RcAlb*-PepIII does not have the potential to penetrate cell membranes, nor allergenic, hemolytic, and toxic potential (Table 1). Regarding Kalata b1, all these biological features were positive, including the high toxicity. The analyses revealed that both peptides possess a cleavage site to enzymes such as trypsin and pepsin (pH 1.3, and pH > 2). The bioinformatics tools also indicated that *RcAlb*-PepIII has high stability to proteolysis in the intestinal environment, considering that the half-life was 2.21 s. Kalata b1 presented normal stability of 0.961 s. *RcAlb*-PepIII is more stable than Kalata b1 even when considering the presence of pepsin and trypsin cleavage sites in their amino acid sequences (Table 1). That may happen because Kalata b1 has more cleavage sites for pepsin than *RcAlb*-PepIII.

### 3.2. Anthelmintic Assays

**Figure 2:**
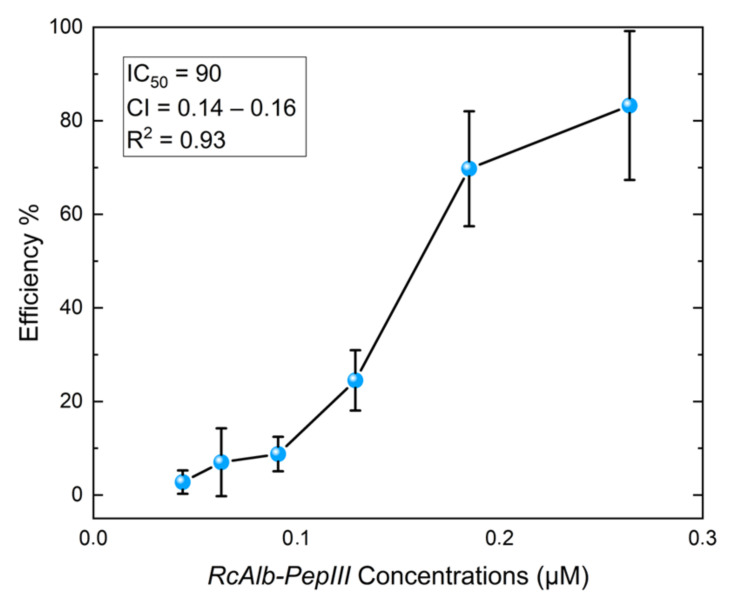
Inhibitory effect of *RcAlb*-PepIII on the development of *Haemonchus contortus*. The graph shows the inhibition of development of *H. contortus* larvae at different concentrations of *RcAlb*-PepIII. L1 and L3 stages were quantified relative to the respective untreated control. Bars represent the means of three independent experiments. Error bars indicate the standard deviations. Half inhibition concentration (IC_50_) in µM, confidence interval (CI), and coefficient of determination (R^2^) were added at the top of the graph.

*RcAlb*-PepIII did not inhibit eggs hatching even at the highest concentration used in this study. However, this peptide had a strong inhibition effect on *H. contortus* larval development with an IC_50_ of 90 µM (Figure 2). The best result was found at 250 µM with inhibition of larval development of 80%. The AFM analysis was employed to gain more insights into the peptide’s action mechanisms based on this activity. 

### 3.3. Atomic Force Microscopy (AFM) Analyses

**Figure 3:**
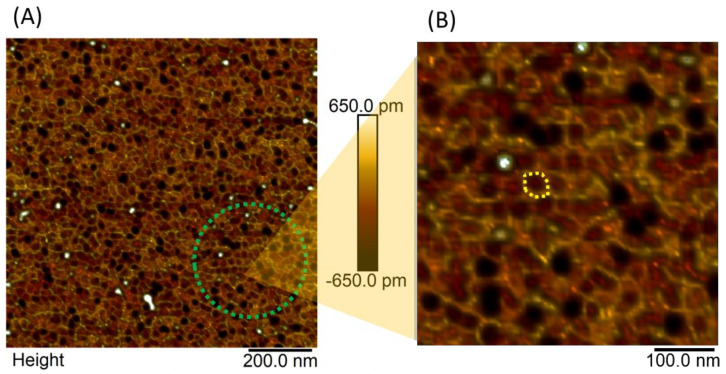
AFM height image of *RcAlb*-PepIII film on mica. (**A**) A chain of peptides (green line circle) that overlaps over the film of the peptide solution. (**B**) The organization of the peptides in the solution film, the yellow detail shows a hexagonal type of structure like a honeycomb.

Given the potential of the AFM technique, it was also employed to characterize the *RcAlb*-PepIII. The AFM analysis shows the organization pattern of the peptides in the solution film deposited over fresh cleaved mica. This pattern is shown mainly in the hexagonal shape, which is like honeycombs (Figure 3A green circle), with differences in the height scale compatible with molecular structures (≈13 Å). Zooming in on the image to 100 nm resolution (Figure 3B yellow dotted shape) shows this pattern in more detail. The yellow dotted figure on one of these structures shows this hexagonal shape.

**Figure 4:**
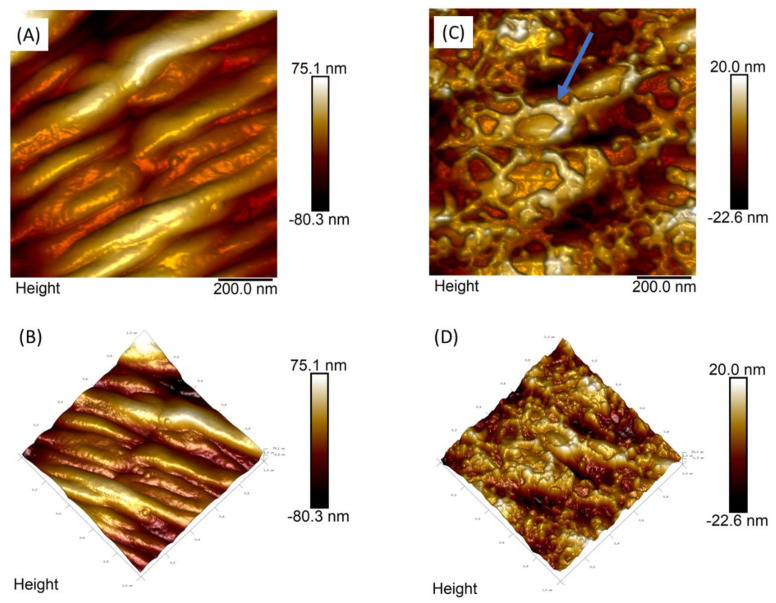
Two-dimensional (2D) and 3D analyses of the L2 cuticle of *H. contortus* by AFM. Non-treated *H. contortus* AFM height image in L2 stage (**A**) and its 3D visualization (**B**), *H. contortus* L2 treated with *RcAlb*-PepIII (**C**) and its three-dimensional visualization (**D**). In the images (**C**,**D**), it is possible to observe the alteration promoted by the attachment of the peptide solution to the cuticle in the L2 nematode. The blue arrow points to the presence of structures on the nematode surface observed after their exposure to peptide solution. Both images have a 1 micrometer square scan.

Figure 4 shows two-dimensional (2D) (Figure 4A,C) and three-dimensional (3D) (Figure 4B,D) topographic images of AFM over *H. contortus* samples in L2 treated (Figure 4C,D) and untreated (Figure 4A,C) with *RcAlb*-PepIII. In Figure 3D and Figure 4C, it is possible to observe the topographic changes promoted by the attachment of the *RcAlb*-PepIII to the cuticle in the L2 stage. From the height scale bars, we observed a marked difference: for samples in the L2 stage without treatment (Figure 4A), the difference in height map is 155.4 nm, while for the map obtained in the sample after treatment, the difference between the structures’ heights on the sample is 42.6 nm (Figure 4C), suggesting wear on the nematode cuticle surface. The blue arrow (Figure 4C) points out structures on the nematode surface only observed after treatment with *RcAlb*-PepIII peptides, which was suggested to be the peptide film not yet absorbed by the cuticle. It is also possible to observe a change in the surface roughness. For control samples (Figure 4A), the mean square roughness (Rq) value was 20.6 nm. In contrast, in *RcAlb*-PepIII-treated samples (Figure 4C), the Rq value is three times lower, 6.11 nm. This significant reduction may be associated with damage to the *H. contortus* cuticle (wear) due to the *RcAlb*-PepIII peptide effect.

**Figure 5:**
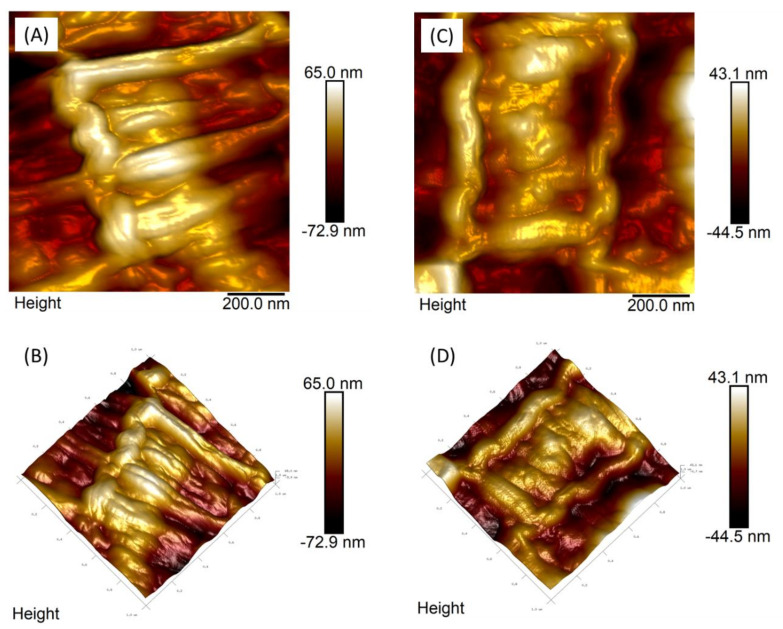
Two-dimensional (2D) and 3D analyses of L2 cuticle of *H. contortus* by AFM. Non-treated *H. contortus* AFM height image in the L3 stage (**A**) and its 3D visualization (**B**), *H. contortus* L3 treated with *RcAlb*-PepIII (**C**) and its 3D visualization (**D**).

The effect of *RcAlb*-PepIII on *H. contortus* L3 was also verified (Figure 5) by two-dimensional (Figure 5A,C) and 3D (Figure 5B,D) topographic images of AFM over *H. contortus* samples in the L3 phase untreated and treated with *RcAlb*-PepIII. Samples in the L3 phase treated with *RcAlb*-PepIII peptides (Figure 5C,D) did not present the structures observed in L2 after treatment (Figure 4C,D), but again, a reduction in the structure height present in the maps after the treatment is observed. For L3 stage control samples, the height difference is 137.9 nm, whereas for samples in the L3 stage treated with the *RcAlb*-PepIII, this difference decreases to 87.6 nm. Differences were also noticed in the Rq values. The Rq value of the surface for the untreated L3 (Figure 5A) was 21.7 and 13.9 nm for treatment with *RcAlb*-PepIII (Figure 5B). These results suggest that at this stage of development, the cuticle also undergoes a wear process. The clear (or not) observation of peptide film in treated samples may be associated with the structure of the nematode in these two stages of development: between the L2 and L3 phase, the formation of the sheath occurs, which in fact changes the structure and probably the mechanism through which it will interact with the peptide.

**Figure 6:**
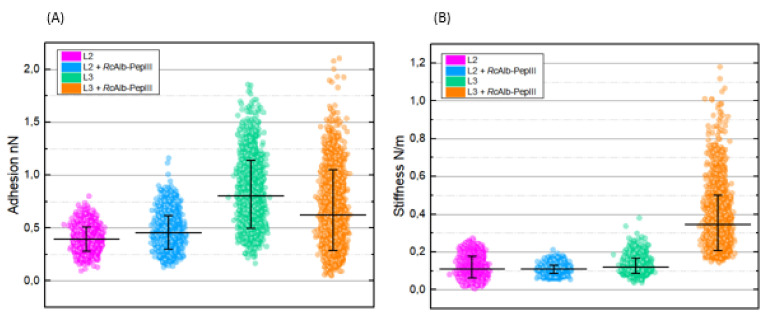
Quantitative adhesion (**A**) and stiffness (**B**) data on *H. contortus* surface in phases L2 and L3 without surface treatment and treated with *RcAlb*-PepIII. For each sample, 65,536 force curves were obtained, each curve providing corresponding adhesion and stiffness values. The higher number of points analyzed were used to produce the standard deviation.

Figure 6 shows adhesion and stiffness graphs of *H. contortus* cuticles for samples untreated and treated with *RcAlb*-PepIII in both L2 and L3 stages. For each sample, 65,536 force curves were obtained, each curve providing a corresponding adhesion and stiffness value (Figure 6A,B, respectively).

It was observed that for each stage of development, the treatment promoted different effects, increasing the adhesion in the L2 stage (control: 0.39 ± 0.09 nN and treated: 0.45 ± 0.13 nN) and decreasing the adhesion in the L3 stage (control: 0.8 ± 0.23 nN and treated: 0.62 ± 0.3 nN) (Figure 6A).

For stiffness results (Figure 6B), in the L2 stage, the scatter plots suggest greater uniformity of the stiffness values after treatment, with mean values before and after treatment not distinguishable (control: 0.11 ± 0.04 and treated: 0.11 ± 0.01). However, in the L3 stage, after treatment, the cuticle’s stiffness values increase remarkably (control: 0.12 ± 0.03 N/m and treated: 0.34 ± 0.13 N/m). 

### 3.4. Toxicity of RcAlb-PepIII 

**Figure 7:**
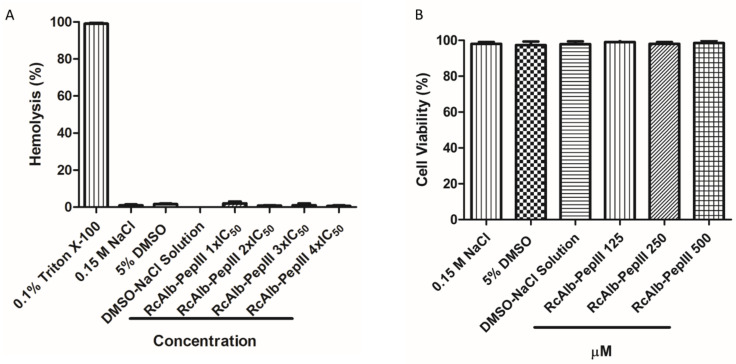
Hemolytic and toxic potential of *RcAlb*-PepIII. (**A**) Hemolytic action of *RcAlb*-PepIII on rabbit red blood cells. It is possible to see no hemolysis even at concentration three times higher than the IC_50_ concentration against *H. contortus*. (**B**) Effect of *RcAlb*-PepII on Vero cells at 500 μM concentration 5.5 times higher than the *RcAlb*-PepIII IC_50_ toward H. contortus. Data were expressed as the mean ± standard deviation of three independent experiments. Different letters indicate significant differences between treatments (*p* < 0.05).

Regarding the toxicity of *RcAlb*-PepIII, bioinformatics analyses revealed no toxicity potential, which was experimentally proven. None of the tested concentrations (Figure 7A) caused the hemolysis of rabbit red blood cells. Here, *RcAlb*-Pep-III was tested at many concentrations as one, two, three, and four times the IC_50_ represented as 1× IC_50_, 2× IC_50_, 3× IC_50_, and 4× IC_50_. Our results revealed that at a concentration four times (4× IC_50_) higher than IC_50_, the peptide has no toxicity of any kind to mammalian cells. Regarding the toxicity to Vero line cells, the concentrations of 125, 250, and 500 µM were not toxic (Figure 7B).

## 4. Discussion

*H. contortus* has developed resistance to all the anthelmintic drug classes, and the development of novel compounds has been slow [1,2,5,6,7,23,24] *H. contortus* and other worms that affect gastrointestinal systems of ruminants are a considerable health concern in livestock production worldwide. *H. contortus*, also known as “barber pole worm”, is a parasite responsible for disease in goats and sheep, leading to economic loss. In the heavy infection caused by *H. contortus*, the worms could consume up to 10% of the host’s blood per day and damage the abomasum, leading to blood plasma loss, hemorrhage, and death [12,13]. 

Various studies have shown that plant source compounds represent a promising alternative to treating helminth/parasitic infections [25,26]. In this group are the cyclotides, which are cyclic antimicrobial peptides purified from plants with anthelmintic potential [11,12,13]. The novelty of this study is the employment of linear peptides toward nematodes. However, all the information produced about anthelmintic peptides is about cyclic peptides. Cyclotides have many biological applications such as anti-HIV, anticancer, insecticidal, and nematicidal activities that have gained the attention of pharmaceutical and agricultural industries to produce new drugs based on peptides [11,12,13]. 

Among the cyclotides, the most famous are Kalata B1 and Kalata B2 purified from the African herb *Oldenlandia affinis* [27]. Both Kalata B1 and Kalata B2 possess activity against several classes of nematodes such as canine and human hookworms *Ancylostoma caninum* and *Necator americanus* [11], against *Schistosoma mansoni* and *Schistosoma japonicum* [28], and *H. contortus* [12]. Kalata B1 and Kalata B2 have an IC_50_ against *H. contortus* of 2.18 and 1.51 µM [12]. Indeed, against *H. contortus*, Kalata B1 and Kalata B2 are 41.3 and 60 times more efficient to reach an IC_50_ than *RcAlb*-PepIII. The anthelmintic molecules may access the target tissues of the nematodes by ingestion or diffusion across the nematode cuticle, which usually is a barrier to drug permeability [29].

The lower activity of *RcAlb*-PepIII compared to Kalata B1 and Kalata B2 is not surprising. However, compared to other molecules, the anthelmintic activity of *RcAlb*-PepIII stands better results. Wanderley et al. [30] presented a cysteine protease from the latex of *Ficus benjamina* that exerts its IC_50_ against *H. contortus* at a concentration of 33 mM. This concentration is 366 times higher than the concentration presented by *RcAlb*-PepIII. Compared to other plant proteins reported by Soares et al. [31], *RcAlb*-PepIII is even stronger. Although they are the most famous, Kalata B1 and B2 are not the most efficient. A family of cyclic peptides from *Viola odorata* (Violaceae) called cycloviolacin are stronger than Kalata peptides. For instance, cycloviolacin O2, O3, and O8 have IC_50_ values against *H. contortus* of 0.12, 0.21, and 0.24 µM [12].

This study is a pioneer in employing AFM analyses to understand the anthelmintic activity mechanism. The AFM technique was useful in the characterization of the *RcAlb*-PepIII films-like structure. AFM analysis in this work was also an efficient tool to verify the affinity of *RcAlb*-PepIII with the cuticle of *H. contortus* in different development stages. AFM analyses revealed changes in the nematode’s cuticle ultrastructure and surface roughness of the L2 stage of *H. contortus*. Those film-like structures or surface wear processes reduce the Rq values (Figure 3, Figure 4, Figure 5 and Figure 6). These are interesting insights about the mechanisms of action of a peptide against nematodes. AFM revealed a mechanism to *Rc*Alb-PepIII which is based on the formation of structures similar to a film, leading to a reduction in the development of larvae in the L2 stage. This mechanism exerted by *RcAlb*-PepIII not involved in membrane lysis could be an advantage compared to cyclotides and may be involved in the non-toxic effects of *RcAlb*-PepIII compared to other peptides. 

For the L3 development stage, although we did not observe the obvious presence of a peptide film on the cuticle surface, changes in the ultrastructure (measured by the height features) and in the surface roughness show the evident structural changes promoted by the treatment. The sheath permeability can be higher in the L3 stage when compared to the L2, which would promote a rapid absorption of the peptides by the surface and make it difficult to observe the film on it. It is necessary to consider that this hypothesis is reinforced by the fact that the same treatment time is employed in both stages. These ultrastructural changes suggest wear of the nematode surface, in both stages of development, corroborating the findings of Svangard et al. [32], which show mechanisms, presented by the other cyclic peptides, that damage and disrupt the membranes [32]. 

It was observed that for each stage of development, the *RcAlb*-PepIII treatment promoted different physical changes, increasing the adhesion in the L2 stage and decreasing the adhesion in the L3 stage. It is important to notice that adhesion forces are a combination of electrostatic, van der Waals, capillary, and escape forces promoted by the chemical bonds [33]. Especially in cases of non-functionalized probes (as done in this research), the adhesion forces are taken as nonspecific interactions, and it is not possible to separate the contribution of each of these forces. However, as probes were employed here to analyze, all samples are made of the same material (Si) and have the same specifications (model, tip radius, etc.), and the experiments for all samples were performed under the same conditions (temperature and air humidity); differences in the adhesion forces between the control and *RcAlb*-PepIII treated samples can means changes in the physical properties of the sample cuticle, indicating the presence of the peptide on the cuticle and/or physical properties changes of its structures due to wear.

For stiffness results, in the L2 stage, the scatter plots suggest greater uniformity of the stiffness values after treatment. However, in the L3 stage, after *RcAlb*-PepIII treatment, the cuticle’s stiffness values increase. This result is promising because at this development stage, the nematode has a sheath, which must be removed to promote its infection stage (L3 unsheathed) [21]. A greater stiffness of this structure can lead to a difficulty for the nematode to progress to the next stage, interrupting, therefore, its life cycle.

Apart from the physicochemical and structural similarities shared by *RcAlb*-PepIII and Kalata B1, the mechanism of anthelmintic activity is entirely different. It must be noticed as an exciting point in the mode of action of *RcAlb*-PepIII. The AFM analysis of *RcAlb*-PepIII showed that it organizes itself in a hexagonal shape forming circular structures similar to honeycombs (Figure 3B yellow dashed circle). The same structures were seen in the larvae treated with *RcAlb*-PepIII (Figure 4B,D). The cuticles of the larvae treated with *RcAlb*-PepIII were covered with a film-like structure that has the same hexagonal shape presented by *RcAlb*-PepIII alone. 

Indeed, the cyclic peptides presented here are stronger than *RcAlb*-PepIII. However, they all share a problem, cytotoxicity, with human cells [32]. Kalata B1 and Kalata B2 are toxic to erythrocytes, fibroblasts, and peripheral blood mononuclear cells [34]. For example, at a concentration of 5 µM, Kalata B1 and Kalata B2 induced 40 and 25% of hemolysis in human erythrocytes, respectively [34]. Regarding cycloviolacin peptides, they also showed cytotoxicity to human cells [32]. Kalata and cycloviolacin peptides share one characteristic. All of them can disintegrate membranes, either from pathogens or from human cells. In cycloviolacin peptides, they needed only five minutes to disintegrate the cell membrane [32]. Despite the good anthelmintic activity, those peptides’ toxicity is a considerable problem for application as drugs. 

In that matter, *RcAlb*-PepIII has an advantage compared to cyclic peptides. Despite having a higher IC_50_ than the cyclotides above cited, *RcAlb*-PepIII presented no toxicity to mammalian erythrocytes and Vero line cells at concentration four times higher than IC_50_ *RcAlb*-PepIII (Figure 7), suggesting a potential application in the treatment of infection caused by *H. contortus* in small ruminants. As it stands, *RcAlb*-PepIII has an IC_50_ of 90 µM against *H. contortus* (Figure 2). 

## 5. Conclusions

Here, we showed for the first time the anthelmintic activity of a synthetic linear peptide against *H. contortus*. AFM analyses of the peptide alone and in contact with the cuticle of L2 provided new insights about the anthelmintic activity mechanism displayed by a peptide, which was not described before. The utmost importance is that *RcAlb*-PepIII did not present any toxic effect on mammalian cells, even at a concentration of five times the IC_50_. Altogether, the results presented here come up with the biotechnological potential of *RcAlb*-PepIII in control of *H. contortus* without any collateral effects to the hosts.

## Figures and Tables

**Table 1:** Physicochemical characterization and in silico analysis of synthetic peptide *RcAlb*-PepIII.

Properties	*RcAlb*-PepIII	Kalata B2
Physicochemical		
Sequence	AKLIPTIA	GLPVCGETCFGGTCNTPGCSCTWPICTRD
^1^ Calculated molecular mass (Da)	826.05	2979.81
^2^ Isoeletric point (pI)	8.80	4.71
^1^ Hydrophobicity	0.864	0.634
^1^ Net charge	+1	−1
^4^ Ramachandran Plot (%)	98	34
^5^ Tm	0.56	-
^5^ sOPEP	−5.73	-
Biological Properties		
^6^ CPP	No	Yes
^7^ Allergic potential	No	Yes
Hemolytic potential	No	Yes
Toxic potential	No	Yes
^9^ Cleavage sites		
Trypsin (high pH)	1	1
Pepsin (pH 1.3)	1	3
Pepsin (pH > 2)	1	4
^10^ Half-life time	2.21	0.961
^10^ Stability	High	Normal

^1^ Calculated using the Antimicrobial Peptide Database. ^2^ Calculated using the ExPASy ProtParam toll. ^4^ Obtained using Rampage. ^5^ Tm and sOPEP scores were calculated by the PEP-FOLD server. ^6^ Cell-Penetrating Peptide (CPP) capacity was calculated using CellPPD tool. ^7^ Allergic potential was calculated using the Antigenic prediction tool. ^8^ Antimicrobial potential was calculated using the iAMPpred tool. ^9^ Cleavage sites were calculated using the Peptide Cutter tool. ^10^ Half-life time in intestinal environment and stability were calculated using the *HLP* tool.

## Data Availability

The data presented in this study are available on request from the corresponding author.

## References

[1] Besier R.B., Kahn L.P., Sargison N.D., Van Wyk J.A. (2016). The Pathophysiology, Ecology and Epidemiology of Haemonchus contortus Infection in Small Ruminants. Adv. Parasitol..

[2] Emery D.L., Hunt P.W., Le Jambre L.F. (2016). Haemonchus contortus: The then and now, and where to from here?. Int. J. Parasitol..

[3] Kaplana R.M., Vidyashankarb A.N. (2012). An inconvenient truth: Global worming and anthelmintic resistance. Vet. Parasitol..

[4] Vatta A.F., Lindberg L.E. (2006). Managing anthelmintic resistance in small ruminant livestock of resource-poor farmers in South Africa. J. S. Afr. Vet. Assoc..

[5] Howell S.B., Burke J.M., Miller J.E., Terrill T.H., Valencia E., Williams M.J., Williamson L.H., Zajac A.M., Kaplan R.M. (2008). Prevalence of anthelmintic resistance on sheep and goat farms in the southeastern United States. J. Am. Vet. Med. Assoc..

[6] Kotze A.C., Prichard R.K. (2016). Anthelmintic Resistance in Haemonchus contortus. History, Mechanisms and Diagnosis. Adv. Parasitol..

[7] Nixon S.A., Welz C., Woods D.J., Costa-Junior L., Zamanian M., Martin R.J. (2020). Where are all the anthelmintics? Challenges and opportunities on the path to new anthelmintics. Int. J. Parasitol. Drugs Drug Resist..

[8] Zasloff M. (2002). Antimicrobial peptides of multicellular organisms. Nature.

[9] da Costa J.P., Cova M., Ferreira R., Vitorino R. (2015). Antimicrobial peptides: An alternative for innovative medicines?. Appl. Microbiol. Biotechnol..

[10] Marr A.K., Gooderham W.J., Hancock R.E. (2006). Antibacterial peptides for therapeutic use: Obstacles and realistic outlook. Curr. Opin. Pharmacol..

[11] Colgrave M.L., Kotze A.C., Kopp S., McCarthy J.S., Coleman G.T., Craik D.J. (2009). Anthelmintic activity of cyclotides: In vitro studies with canine and human hookworms. Acta Trop..

[12] Colgrave M.L., Kotze A.C., Ireland D.C., Wang C.K., Craik D.J. (2008). The anthelmintic activity of the cyclotides: Natural variants with enhanced activity. Chembiochem.

[13] Colgrave M.L., Kotze A.C., Huang Y.-H., O’Grady J., Simonsen S.M., Craik D.J. (2008). Cyclotides: Natural, Circular Plant Peptides that Possess Significant Activity against Gastrointestinal Nematode Parasites of Sheep. Biochemistry.

[14] Souza P.F.N., Marques L.S.M., Oliveira J.T.A., Lima P.G., Dias L.P., Neto N.A.S., Lopes F.E.S., Sousa J.S., Silva A.F.B., Caneiro R.F. (2020). Synthetic antimicrobial peptides: From choice of the best sequences to action mechanisms. Biochimie.

[15] Souza P.F.N., Vasconcelos I.M., Silva F.D.A., Moreno F.B., Monteiro-Moreira A.C.O., Alencar L.M.R., Abreu A.S.G., Sousa J.S., Oliveira J.T.A. (2016). A 2S albumin from the seed cake of ricinus communis inhibits trypsin and has strong antibacterial activity against human pathogenic bacteria. J. Nat. Prod..

[16] Dias L.P., Souza P.F.N., Oliveira J.T.A., Vasconcelos I.M., Araújo N.M.S., Tilburg M.F.V., Guedes M.I.F., Carneiro R.F., Lopes J.L.S., Sousa D.O.B. (2020). RcAlb-PepII, a synthetic small peptide bioinspired in the 2S albumin from the seed cake of Ricinus communis, is a potent antimicrobial agent against Klebsiella pneumoniae and Candida parapsilosis. Biochim. Biophys. Acta Biomembr..

[17] Sharma A., Singla D., Rashid M., Raghava G.P.S. (2014). Designing of peptides with desired half-life in intestine-like environment. BMC Bioinform..

[18] Thévenet P., Shen Y., Maupetit J., Guyon F., Derreumaux P., Tufféry P. (2012). PEP-FOLD: An updated de novo structure prediction server for both linear and disulfide bonded cyclic peptides. Nucleic Acids Res..

[19] Coles G.C., Bauerb C., Borgsteedec F.H., Geertsd S., Kleie T.R., Taylora M.A., Wallerf P.J. (1992). World Association for the Advancement of Veterinary Parasitology (W.A.A.V.P.) methods for the detection of anthelmintic resistance in nematodes of veterinary importance. Vet. Parasitol..

[20] Demeler J., Küttler I., Samson-Himmelstjerna G. (2010). Adaptation and evaluation of three different in vitro tests for the detection of resistance to anthelmintics in gastro intestinal nematodes of cattle. Vet. Parasitol..

[21] Costa-Junior L.M., Silva C.R., Soares A.M.S., Menezes A.S., Silva M.R.L., Amarante A.F.T., Costa E.F., Alencar L.M.R. (2020). Assessment of biophysical properties of Haemonchus contortus from different life cycle stages with atomic force microscopy. Ultramicroscopy.

[22] Oliveira J.T.A., Souza P.F.N., Vasconcelos I.M., Dias L.P., Martins T.F., Van Tilburg M.F., Guedes M.I.F., Sousa D.O.B. (2019). Mo-CBP3-PepI, Mo-CBP3-PepII, and Mo-CBP3-PepIII are synthetic antimicrobial peptides active against human pathogens by stimulating ROS generation and increasing plasma membrane permeability. Biochimie.

[23] Gilleard J.S., Redman E. (2016). Genetic Diversity and Population Structure of Haemonchus contortus. Adv. Parasitol..

[24] Šimpraga M., Ljubičić I., Hlede J.P., Vugrovečki A.S., Marinculić A., Tkalčić S. (2015). Alternative approaches for the control of gastrointestinal nematodes in sheep farming: A review. Berl. Munch. Tierarztl. Wochenschr..

[25] Alonso-Díazab M.A., Torres-Acostaa J.F.J., Sandoval-Castroa C.A., Capetillo-Leala L., Brunet S., Hostec H. (2008). Effects of four tropical tanniniferous plant extracts on the inhibition of larval migration and the exsheathment process of Trichostrongylus colubriformis infective stage. Vet. Parasitol..

[26] Hernández-Villegasa M.M., Borges-Argáeza R., Rodriguez-Vivasb R.I., Torres-Acostab J.F.J., Méndez-Gonzaleza M., Cáceres-Farfana M. (2011). Ovicidal and larvicidal activity of the crude extracts from Phytolacca icosandra against Haemonchus contortus. Vet. Parasitol..

[27] Jennings C., West J., Waine C., Craik D., Anderson M. (2001). Biosynthesis and insecticidal properties of plant cyclotides: The cyclic knotted proteins from Oldenlandia affinis. Proc. Natl. Acad. Sci. USA.

[28] Malagón D., Botterill B., Gray D.J., Lovas E., Duke M., Gray C., Kopp S.R., Knott L.M., McManus D.P., Daly N.L. (2013). Anthelminthic activity of the cyclotides (kalata B1 and B2) against schistosome parasites. Biopolymers.

[29] Holden-Dye L., Walker R.J. (2014). Anthelmintic drugs and nematicides: Studies in Caenorhabditis elegans. Wormbook.

[30] Wanderley L.F., Soares A.M., Silva C.R., Figueiredo I.M., Ferreira A.T., Perales J., Mota H.R., Oliveira J.T., Costa L.M. (2018). A cysteine protease from the latex of *Ficus benjamina* has in vitro anthelmintic activity against *Haemonchus contortus*. Rev. Bras. Parasitol. Veterinária.

[31] Soares A.M.S., Oliveira J.T.A., Rocha C.Q., Ferreira A.T.S., Perales J., Zanatta A.C., Vilegas W., Silva C.R., Costa-Junior L.M. (2018). Myracrodruon urundeuva seed exudates proteome and anthelmintic activity against Haemonchus contortus. PLoS ONE.

[32] Svangård E., Burman R., Gunasekera S., Lövborg H., Gullbo J., Göransson U. (2007). Mechanism of action of cytotoxic cyclotides: Cycloviolacin O2 disrupts lipid membranes. J. Nat. Prod..

[33] Butt H.J., Cappella B., Kappl M. (2005). Force measurements with the atomic force microscope: Technique, interpretation and applications. Surf. Sci. Rep..

[34] Henriques S.T., Huang Y.-H., Chaousis H., Wang C.K., Craik C.J. (2014). Anticancer and toxic properties of cyclotides are dependent on phosphatidylethanolamine phospholipid targeting. Chembiochem.

